# Vestibular rehabilitation improves spontaneous nystagmus normalization in patients with acute unilateral vestibulopathy

**DOI:** 10.3389/fresc.2023.1122301

**Published:** 2023-05-31

**Authors:** Michel Lacour, Christophe Lopez, Alain Thiry, Laurent Tardivet

**Affiliations:** ^1^ Aix-Marseille University, CNRS, Laboratory of Cognitive Neuroscience (LNC), FR3C, Marseille, France; ^2^ Independent Researcher, Fuveau, France; ^3^ Private Practitioner, Nice, France; ^4^ Otorhinolaryngology Department, CHU Nice, Nice, France

**Keywords:** acute unilateral vestibulopathy, spontaneous nystagmus, vestibular rehabilitation, unidirectional rotation paradigm, early vs. late vestibular rehabilitation

## Abstract

**Introduction:**

Spontaneous nystagmus (SN) can be observed after acute unilateral vestibulopathy (AUVP). The slow phase eye velocity of the SN progressively decreases in darkness as the result of rebalanced neurophysiological activity between both vestibular nuclei, a process that can take several months. Although this compensatory process can occur spontaneously, there is poor evidence that vestibular rehabilitation (VR) can facilitate the process.

**Methods:**

We documented the natural time course of SN reduction in patients with AUVP, as well as the effects of VR by means of a unilateral rotation paradigm. In a retrospective study (Study 1: *n* = 126 AUVP patients), we compared the time course of the SN reduction in patients with VR (*n* = 33) and without VR (*n* = 93). In a prospective study (Study 2: *n* = 42 AUVP patients), we compared the effects of early VR (*n* = 22; initiated within the first two weeks of symptoms onset) or late VR (*n* = 20; initiated after the second week of symptoms onset) on the time course of the SN reduction.

**Results:**

Study 1 showed shorter median time of SN normalization in patients with VR compared to patients without VR (14 days and 90 days, respectively). Study 2 showed that AUVP patients with early and late VR had a similar median time of SN normalization. The SN slow phase eye velocity was significantly decreased as early as the end of the first VR session in both groups, and kept decreasing at each subsequent VR session. In the early VR group, 38% of the patients had slow phase eye velocity below 2°/s after the first VR session, 100% after the fifth session. Similar findings were observed in the late VR group.

**Discussion:**

Taken together, these results indicate that VR with a unidirectional rotation paradigm speeds up the normalization of SN. This effect seems independent of the time between symptoms onset and commencement of VR, but early intervention is recommended to speed up the SN reduction.

## Introduction

The vestibular syndrome observed in patients with an acute unilateral vestibulopathy (AUVP) comprises both static symptoms observed when patients are stationary, and dynamic symptoms when the patients' head or whole body is moved (1). Among the static symptoms is the ocular-tilt reaction, which combines oculomotor signs (spontaneous nystagmus, skew deviation, eye cyclotorsion), postural signs (head and body tilt to the side of AUVP), and perceptual signs (vertigo, tilt of the subjective visual vertical to the side of AUVP). Static symptoms are almost fully compensated after AUVP through the process of vestibular compensation. Vestibular compensation is a spontaneous, or “naturalistic”, functional recovery after damage to the peripheral vestibular system, and is among the best documented postlesional plasticity phenomena, coming to be recognized as the “neuro-otologist’s best friend” (2).

Animal models of unilateral vestibular loss showed that static symptoms result from a neurophysiological imbalance between the ipsilesional and contralesional vestibular nuclei, with decreased resting activity in the neurons on the ipsilesional side and near normal resting activity on the contralesional side [unilateral labyrinthectomy in guinea pigs (3–6); unilateral vestibular neurotomy in cats (7)]. The spontaneous firing rate and sensitivity of the Type I vestibular neurons in the ipsilesional vestibular nuclei is further reduced by an increased inhibitory drive from the intact, contralesional side through commissural pathways. Compensation of these deficits in animal models is well documented and there is a general agreement that the recovery of a balanced neurophysiological activity in the vestibular nuclei is a key compensatory mechanism. The Bechterew phenomenon, that is, the mirror image of the static symptoms observed in compensated animals when the intact labyrinth is destroyed, was the first evidence of restored spontaneous activity in the deafferented vestibular nuclei (8). A combination of molecular, cellular, and sensory substitution mechanisms contributes to restore neuronal activity in the ipsilateral vestibular nuclei (9, 10).

Animal models of unilateral vestibular loss also indicate that the compensation of static symptoms has a time course similar to that of the recovery of a balanced resting discharge in the vestibular nuclei. The recovery of balanced neurophysiological activity in the vestibular nuclei after acute unilateral peripheral vestibular loss is quicker in rodents [1 week (9)] than in cats [6 weeks (10–12)]. However, time frames for AUVP patients are poorly documented. For example, head and trunk orientation as well as trunk stabilization in the roll plane are still altered three months after unilateral vestibular neurectomy (13). Other studies have noted that one year is required for the subjective visual vertical and horizontal to be fully compensated (14, 15), and for body roll-tilt perception to normalize (16). Ocular cyclotorsion is another static oculomotor sign still observed at least 3 to 6 months after unilateral vestibular neurectomy (17), suggesting that it could be a permanent otolithic problem (18). Thus, when compared to animal models, static deficits in humans are compensated within a much longer time period.

Spontaneous nystagmus (SN) in AUVP patients consists of slow horizontal and torsional eye deviations (slow phases) toward the affected side interrupted by fast eye movements (quick phases) away from the affected side. To an observer, this SN shows both eyes beating away from the affected side (18). SN is temporary and resolves or decreases on its own with time. It is reduced or suppressed by visual fixation but shows high inter-individual variability when recorded in the light. Fushiki et al. (19) showed that about 50% of the patients exhibited SN in the light on the third day after symptom onset, and 20% of the patients had SN on the eighth day. Furthermore, the recovery time of the SN increased with the prevalence of canal paresis (19). SN recorded in total darkness showed a much longer recovery time, ranging from several weeks to months after symptoms onset. For some patients, a small SN persists in darkness as a permanent legacy of their unilateral vestibular loss, suggesting an incomplete recovery of neurophysiological activity in the vestibular nuclei long after the vestibular loss (18).

Vestibular rehabilitation (VR) is recognized today as a safe and effective way to accelerate and promote functional recovery (20–25), but there is a paucity of data on how VR can impact the time course of the SN reduction [for other parameters, see (26)]. This is of high clinical relevance since faster SN reduction is expected to have positive benefits at a behavioral level [i.e., stability; see (1)] and for the patients' quality of life.

**Figure 1:**
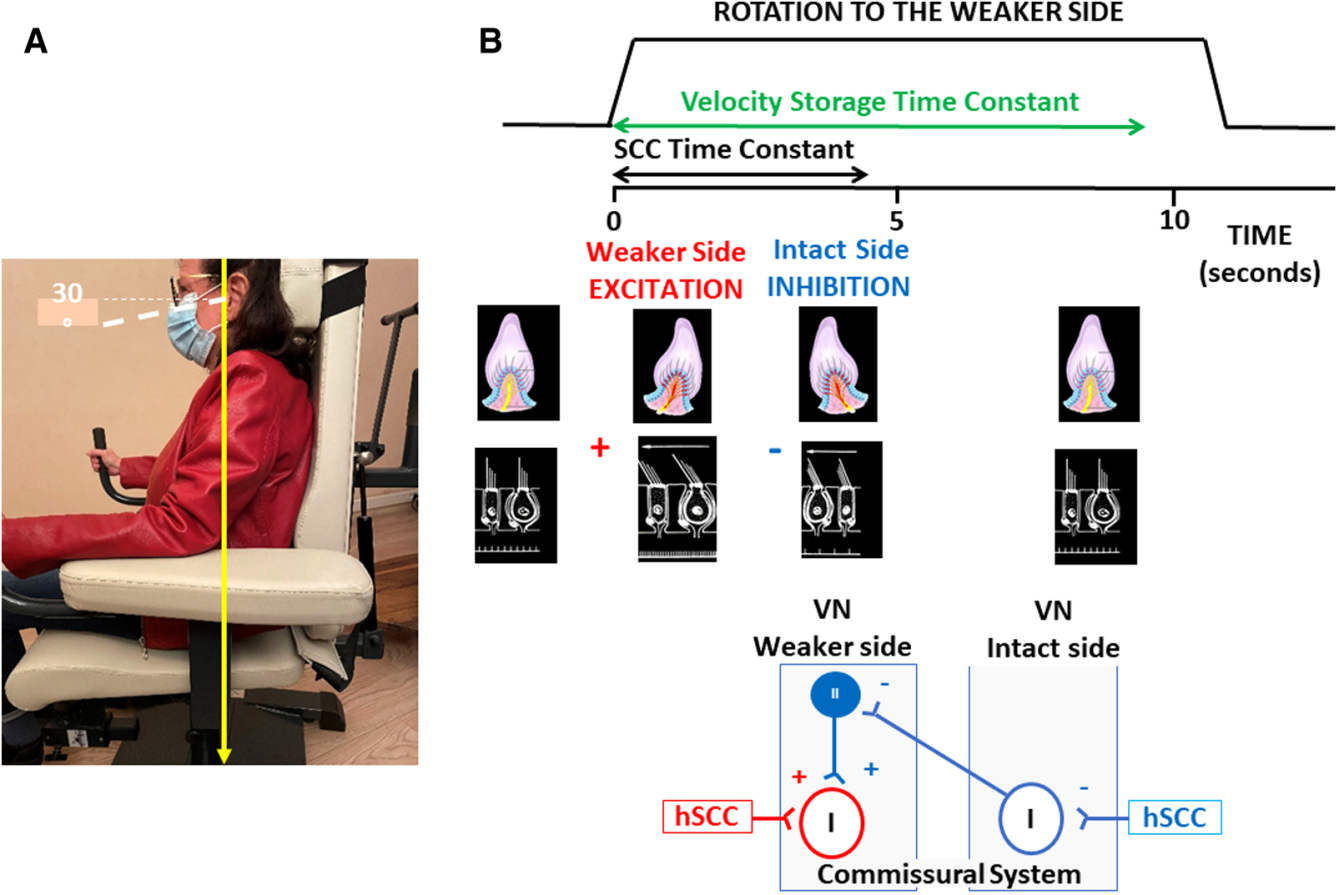
Unidirectional rotation paradigm and hypothetical mechanisms underlying its therapeutic effect. (**A**) Illustration of the rotating chair and position of the patient's head and body, with eyes closed. (**B**) From top to bottom: Schematic drawings of the velocity profile of the stimulation, the induced ampullofugal and ampullopetal flows in the ipsilateral and contralateral horizontal semicircular canals, and the hypothetical mechanisms within the vestibular nuclei. The velocity profile is for five full turns at 200°/s to the weaker side, and shows estimated values of the time constants of the cupula (in black: ∼4 s) and of the velocity storage mechanisms that prolongs the patient's perception of rotation (in green: ∼9 s). The displacement of the cupula is excitatory on the weaker side and inhibitory on the intact side. The hypothetical mechanisms of compensation in the vestibular nuclei are illustrated in the bottom diagrams. Unidirectional rotation to the affected side stimulates remaining intact vestibular afferents projecting to second order neurons (Type I neurons) on the weaker side, and inhibits the Type I neurons on the intact side. Type I neuronal inhibition deactivates the inhibitory drive of Type II neurons on the affected side through the commissural pathways and induces a disinhibition of the Type I neurons on the weaker side. These two mechanisms could restore the spontaneous activity of the second order vestibular neurons on the weaker side, and rebalance the resting discharge of the vestibular nuclei on both sides. hSCC, horizontal semicircular canal; VN, vestibular nuclei; I, Type I second-order vestibular neurons; II, Type II inhibitory vestibular neurons;+indicates a faciliatory effect (in red) or a disinhibitory effect (in blue);−indicates an inhibitory or a de-facilitating effects.

The present study aimed at determining whether VR using a unidirectional rotation protocol influences the time course of the SN reduction recorded in darkness in AUVP patients. This rehabilitation method, which consists of rotating the patient's whole body in the yaw plane towards the hypofunctioning side, was first proposed at the end of the XX^th^ century by Alain Semont, a French physiotherapist, as a clinical tool to reduce acute vestibular asymmetries. This VR protocol is still used by French physiotherapists, but its effectiveness has never been supported by peer-reviewed publications, and has been ignored in most other countries. Only one article, published in French, reported subjective and objective improvements of posture and balance in Menière's disease patients treated with rotational exercises, albeit for reasons that remain to be clarified (27). More recently, we reported positive outcomes of the protocol on postural recovery (28, 29) and dynamic horizontal canal function (30) in AUVP patients. We postulated that unilateral rotations in darkness to the weaker side could reduce the imbalance in spontaneous activity of the bilateral vestibular nuclei by restoring the resting discharge on the weaker side using two complementary mechanisms: stimulation of remaining intact vestibular afferents on the affected side, and inhibition of the intact side that reduces the commissural inhibition exerted by the intact side on the injured side (Figure 1).

The impact of VR with a unidirectional rotation paradigm on the time course of the SN reduction was analyzed in patients with VR and without VR (Study 1), and in in patients who underwent VR at different stages after symptoms onset (Study 2), to determine whether there are any benefits of VR and of early intervention.

## Materials and methods

### Participants

Clinical examination and patient's history was done by LT (author LT) and used to diagnose AUVP. All AUVP patients exhibited the five main inclusion criteria proposed by Strupp and Magnusson (31): acute onset of spinning vertigo, horizontal rotatory SN beating to the intact side, a positive head impulse test (HIT) on the weaker side, nausea, and postural imbalance. The pathological weaker side was determined when the angular vestibulo-ocular (aVOR) gain during passive video HIT (vHIT Ulmer, Synapsis, Marseille, France) was below 0.70 and when overt/covert saccades were observed. Horizontal aVOR gain on the intact side above 0.80 was required for patient inclusion. Positional vertigo, central vestibular pathology, ocular motor dysfunctions, and drug treatment were exclusion criteria. Vestibular deficit was documented on the basis of the HIT for the lateral, anterior and posterior canals. Caloric vestibular testing was not systematically done due to discomfort, but was always pathological on the weaker side when performed.

All patients provided written informed consent to participate and were asked to abstain from antivertigo drugs for the duration of the study.

#### Study 1: time course of the SN reduction with and without VR

Study 1 is a retrospective analysis that focused on the slow phase eye velocity (SPEV) of the SN measured in darkness. The compensation time course was evaluated in 92 patients who did not undergo VR with a unidirectional rotation paradigm and in 33 patients who did (see Table 1 for patients' characteristics).

**Table 1:** Characteristics of the AUVP patients in retrospective Study 1.

	Without VR	With VR
*n*	92	33
**Sex (*n*)**		
Males	44	17
Females	48	16
**Age (years)**		
Mean ± SD	60.5 ± 14.5	57.7 ± 12.0
Range	18–82	30–75
**Side of hypofunction (*n*)**		
Left ear	45	14
Right ear	47	19
**Time from symptoms onset (days)**		
Mean ± SD	17.3 ± 16.7	7.2 ± 2.7
Range	2–90	4–13

The non-rehabilitated group included AUVP patients whose SPEV was measured at their initial visit, which took place 2–90 days after symptoms onset.

The rehabilitated group included patients whose initial visit took place 4–13 days after symptoms onset. Their initial visit was the day of study inclusion and the day of the first VR session. Patients received 3–9 VR sessions and the SPEV was measured at each visit. The effect of VR was assessed for each patient by comparing the SPEV of the SN recorded before and immediately after each VR session.

#### Study 2: time course of the SN reduction with early and late VR

Study 2 is a prospective analysis of the SPEV of the SN recorded in darkness in 42 AUVP patients who underwent VR with a unilateral rotation paradigm. VR was performed early (*n* = 22; initiated within the first two weeks) or late (*n* = 20; initiated after the second week) after symptoms onset (see Table 2 for patients' characteristics). The effect of VR was assessed for each patient by comparing the SPEV of the SN recorded before and immediately after each VR session. Study 2 included supplementary measurements of the static and dynamic subjective visual vertical (SVV) and the Dizziness Handicap Inventory (DHI) score.

**Table 2:** Characteristics of the AUVP patients in prospective Study 2.

	Early VR	Late VR
*n*	22	20
**Sex (*n*)**		
Males	11	11
Females	11	9
**Age (years)**		
Mean ± SD	62.8 ± 15.0	62.5 ± 14.5
Range	35–86	35–82
**Side of hypofunction (*n*)**		
Left ear	12	10
Right ear	10	10
**Time from symptoms onset (days)**		
Mean ± SD	6.9 ± 2.3	35.9 ± 9.5
Range	2–13	16–42

### Assessment of vestibular deficit

HIT was performed with passive head rotation to the healthy and weaker sides in seated patients. Head rotations were done with 10° peak amplitude, 200°/s peak velocity and ∼2,000°/s^2^ peak acceleration. Recording of the aVOR of the horizontal canals was done by tilting the patient's head downwards by 30° to place the lateral semicircular canals in the horizontal plane. Recordings of the aVOR of the anterior and posterior canals were done by turning the patient's head 45° to the right and to the left. HIT was performed randomly to elicit unpredictable timing and direction of head movement. Gain values of the aVOR were approximated by the Synapsis software as the ratio:

$$\mathrm{Gain}=\frac{\mathrm{peak}\;\mathrm{eye}\;\mathrm{velocity}}{\mathrm{peak}\;\mathrm{head}\;\mathrm{velocity}}$$

An average gain value was calculated before and after VR from 5 correctly performed tests on the intact and weaker sides. However, more than 5 trials were generally done due to blinks or imperfect target fixation.

### Assessment of the spontaneous nystagmus

The SN was recorded using videonystagmography (Framiral, Grasse, France) in patients seated with their head pitched 30° downwards to a position in which the lateral semicircular canals were placed in the horizontal plane. Patients were instructed to keep their eyes open in the video headset and to look straight ahead. SN was recorded in darkness for 30 s and the mean SPEV was calculated. SN was recorded before and after each VR session (Study 1 and Study 2), and at the moment of the inclusion visit for the patients without VR (Study 1).

### Vestibular rehabilitation with the unidirectional rotation paradigm

The rationale of VR with a unidirectional rotation paradigm is to reduce the vestibular asymmetry by simultaneously stimulating the weaker side and inhibiting the intact side. We postulated that unilateral rotations in darkness could reduce the imbalance in the spontaneous neuronal activity of the bilateral vestibular nuclei by restoring the spontaneous firing rate of the second-order vestibular neurons on the weaker side. Rotation to the weaker side (a) inhibits Type I neurons on the intact, contralateral side, which disinhibits the Type I neurons on the weaker side by means of the commissural pathways, and (b) stimulates remaining intact vestibular afferents contacting the Type I cells on the weaker side. Both mechanisms would act jointly to restore the spontaneous discharge in the vestibular nuclei on the affected side, and rebalance the spontaneous resting discharge on both sides. Figure 1 illustrates these hypothetical mechanisms resulting from the rotation-induced vestibular stimulation. A facilitated normalization of the SN after VR could be seen as the therapeutic effect of the unidirectional rotation paradigm. The protocol was performed in darkness to avoid possible visuo-vestibular interactions that were not investigated in the present study. Even though sensory substitution using visual cues is involved in the compensation of the static vestibular deficits, recovery of SN is not dependent on visual inputs (32). SN decreases at the same rate in animals kept in the dark immediately after unilateral labyrinthectomy as in animals kept in a lighted environment.

The physiotherapist (author AT) performed all VR sessions. The unidirectional rotation paradigm consisted of whole-body passive rotations to the patient's weaker side using a rotating chair (Framiral, Grasse, France). Patients were seated with their eyes closed and head tilted 30° downwards to place the horizontal semicircular canals close to the horizontal, and rotated during a minimum of three full 360° turns at high velocity (200°/s, 2,000°/s^2^). Patients who tolerated stimulation underwent a higher number of turns. The chair was suddenly stopped at the end of the last lap. Patients were then asked to keep their eyes closed and to indicate verbally when their sensation of rotation in the opposite direction was over (vection protocol), or to open their eyes and fixate a visual target until the illusory target motion stopped (fixation protocol). The two protocols provided comparable data used to test the habituation of the intact labyrinth after repetition of the rotations and of the training sessions (data not reported here; manuscript in preparation). This is the subject of ongoing experiments aimed to better understand the therapeutic effect of the unidirectional rotation protocol. When the post-rotatory nystagmus had disappeared, and after 1 min of rest, another series of chair rotations was performed. A minimum of three series of chair rotations were made during the same VR session and up to ten series of rotations were made if tolerated by the patient, with a total duration that did not exceed 30 min. Participants completed VR sessions until the SN recorded in darkness had a SPEV below 2°/s. VR sessions were done twice a week for four weeks after inclusion. SN with SPEV below 2°/s were considered non-pathological and used to evaluate the percentage of patients who recovered over time.

### Supplementary measurements

In addition to SPEV of the SN, Study 2 analyzed perceived vestibular handicaps using the French adaptation of the Dizziness Handicap Inventory (DHI) (33) before and after VR. The total score incorporates 25 physical, functional and emotional items rated on a three-point scale, with 4, 2 and 0 corresponding to the answers “yes”, “sometimes” and “no”, respectively. The total DHI score ranges from 0 to 100. Patients with AUVP have generally moderate handicap with DHI scores ranging from 40 to 60 (29, 30, 34).

The perception of the static and dynamic subjective visual vertical (SVV) was also measured in Study 2 at the beginning of VR and immediately at the end of the last VR session. Patients were standing upright and faced a screen 1 m in front of them, at eye level. They wore goggles narrowing their visual field to the intended visual scene on which a red laser line was projected (Framiral, Grasse, France). The line was positioned randomly at ±15° or ±30° relative to the true gravitational vertical and patients were asked to rotate the line clockwise or counterclockwise using two handheld pushbuttons until they aligned the laser line with their perception of verticality. The static SVV was measured binocularly in darkness. Five trials were carried out for each initial positioning of the line and the mean orientation was calculated.

The dynamic SVV was measured with the same device, but with a random visual pattern made of white dots of different sizes rotating clockwise or counterclockwise at 20°/s. Patients were asked to adjust the laser line to the vertical during the visual scene rotation. In healthy participants clockwise and counterclockwise rotations result in symmetrical tilt of the SVV up to 10–15° in the direction of the visual field rotation (15) and, therefore, there is no directional preponderance. The directional preponderance of the dynamic SVV (15) in the AUVP patients was calculated as:

$$\mathrm{Directional}\;\mathrm{preponderance}=\frac{\mathrm{ipsilateral}\;\mathrm{SVV}\text{–}\mathrm{contralateral}\;\mathrm{SVV}}{\mathrm{ipsilateral}\;\mathrm{SVV}+\mathrm{contralateral}\;\mathrm{SVV}}\times 100.$$

Average values were calculated over three trials presented in a randomized order on each side.

### Statistical analyses

#### Linear mixed-effects model

Changes in the SPEV of the SN over time was analyzed with a linear mixed-effects model using log transformed SPEV values [i.e., log_10_(SPEV + 1)]. This was to improve the normality of the SPEV distribution, to be able to perform linear regressions, and to account for repeated measures for patients tested during several VR sessions. For Study 1, we used Participants as random effects and Session (number of days after symptoms onset, coded as a covariate in the model), Rehabilitation (with VR vs. without VR), and interaction of Session × Rehabilitation as fixed effects. For Study 2, we used Participants as random effects and Session (number of days after the first VR session, coded as a covariate in the model), Timing of VR (early VR vs. late VR), and interaction of Session × Timing of VR as fixed effects. The analyses were conducted using SPSS version 28.0 (IBM). *p* values <0.05 were considered statistically significant.

#### Survival analysis

We also estimated the normalization of the SN (i.e., when SPEV was <2°/s) after symptom onset (for Study 1) or after the first VR session (for Study 2) using the Kaplan–Meier method on non-transformed SPEV values in GraphPad Prism version 9.4.1. Survival curves were plotted and a Log-rank test was calculated to compare SN normalization in AUVP patients without and with VR in Study 1, and to compare SN normalization in patients who underwent early and late VR in Study 2. *p* values <0.05 were considered statistically significant.

## Results

### Study 1: time course of the SN reduction with and without VR

#### Descriptive analysis of the individual curves of SN over time

**Figure 2:**
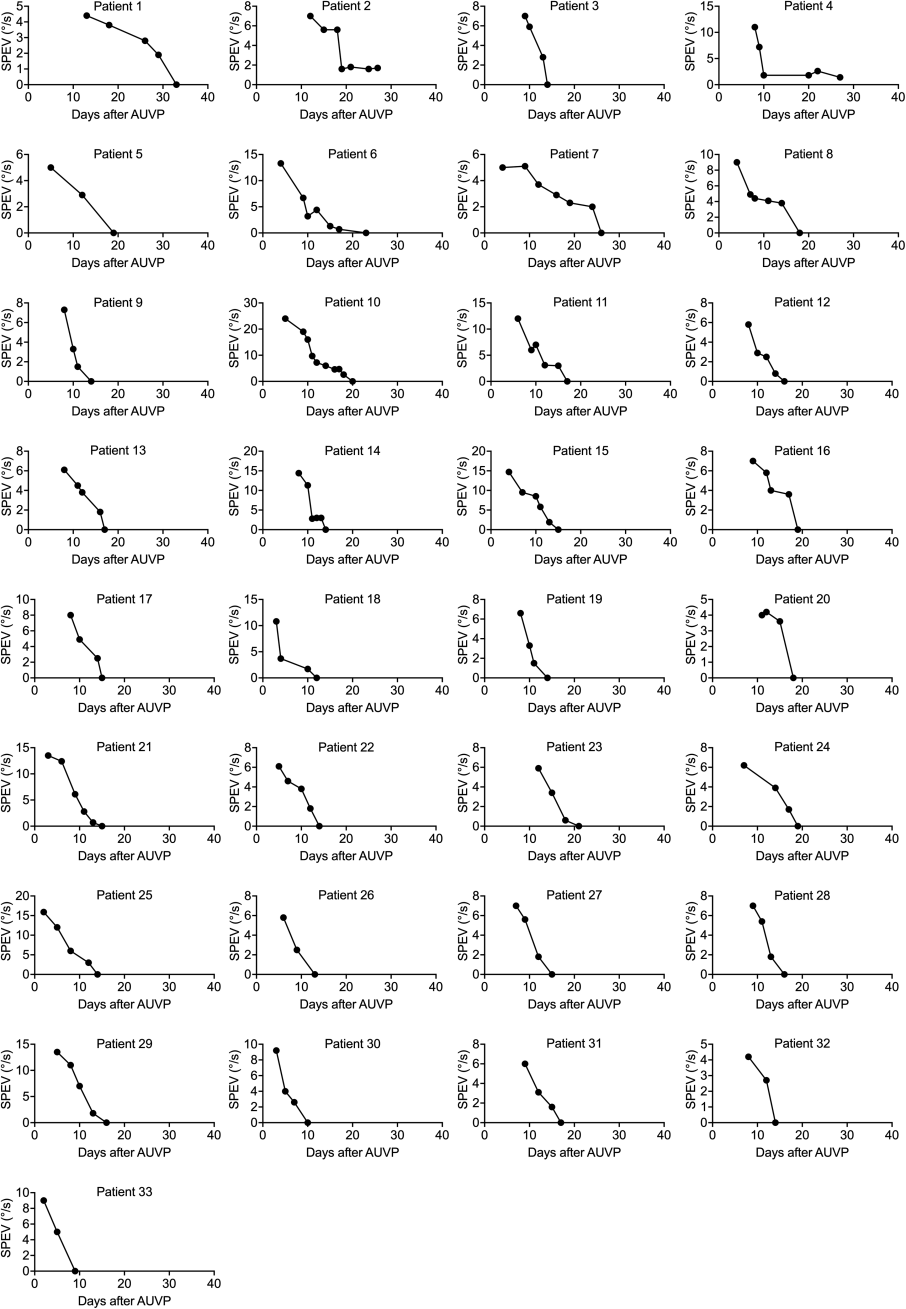
Individual curves of the SPEV of the SN over time in patients who underwent VR (study 1). SPEV of the SN as a function of days after symptoms onset for each patient who underwent VR with the unidirectional rotation paradigm. Two to nine VR sessions were completed to recover a SPEV < 2°/s. All patients recovered to non-pathological SN between 9 and 29 days after symptoms onset.

Figure 2 shows the evolution of the SPEV of the SN in 33 patients who underwent VR. Despite the fact that VR commenced at various durations post AUVP (2–13 days after symptoms onset), all patients had a SN with a SPEV < 2°/s between 9 and 29 days after symptoms onset. Recovery of nystagmus with SPEV < 2°/s involved 2–9 VR sessions.

#### Linear mixed-effects model

**Figure 3:**
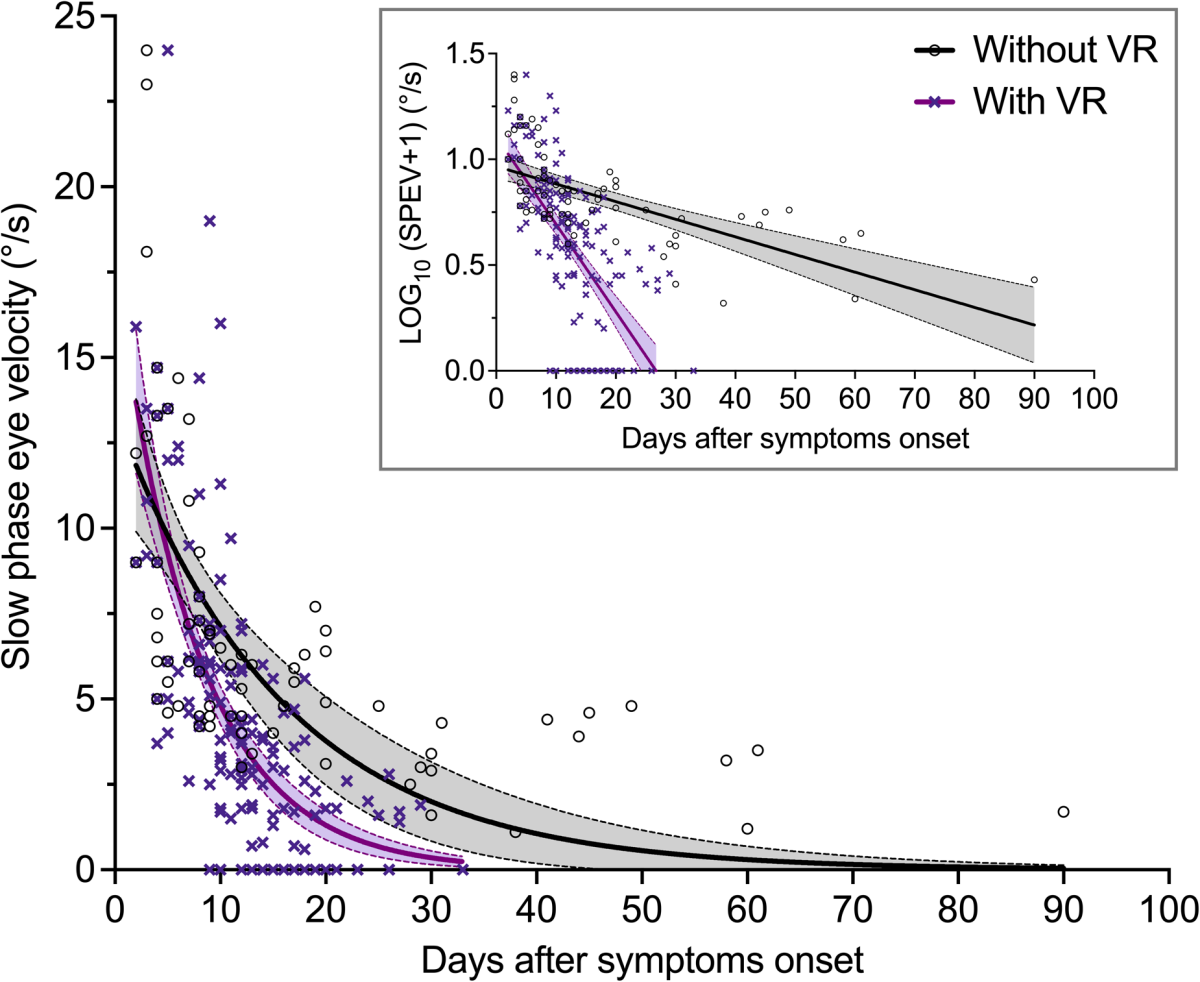
Evolution of the SPEV of the SN over time in patients with and without VR (Study 1). SPEV of the SN as a function of days after symptom onset, and one-phase decay regression curves for both groups of patients. The equation used was: *y* *=* *y*_0 _× exp (−*x*/*τ*), where *y* is the SPEV in °/s, *x* is the time in day, and τ is the time constant in days (goodness of fit: *R*^2^ = 0.40 for patients without VR; *R*^2^ = 0.47 for patients with VR). The filled areas are the 95% confidence interval of parameters. The graph in the inset shows that log-transformation of the SPEV values allows for computation of linear regressions.

A linear mixed-effects model on the log-transformed values of SPEV was used to compare the time course of SN reduction in patients with VR and without VR, accounting for multiple observations per patient for the group receiving VR. The inset in Figure 3 shows that the log-transformation allows for linear regression calculations.

Table 3 shows the parameters estimates for the fixed effects. As expected, the linear mixed-effects model indicated that the log-transformed SPEV values were significantly modulated by the time elapsed since the symptoms onset (*B* = −0.009, SE = 0.002, *p* < 0.001). There was no significant effect of VR (*B* = 0.058, SE = 0.07, *p* = 0.408), indicating no group difference in the severity of the SN at time zero. There was a significant interaction of Session × Rehabilitation (*B* = −0.033, SE = 0.005, *p* < 0.001), indicating a different evolution of SPEV over time as a function of VR. Comparison of the slope of the fit for the log-transformed SPEV values in the group with VR (−0.042) and without VR (−0.009) indicated that the recovery was 4.7 times faster in the group of patients who underwent VR.

**Table 3:** Estimates of fixed effects for Study 1.

Parameter	*B*	SE	*df*	*t*	*p*	95% CI (lower, upper)
Intercept	0.995	0.033	213.55	30.585	<0.001	0.931, 1.059
Rehabilitation^a^	0.058	0.070	229.14	0.830	0.408	−0.080, 0.196
Session	−0.009	0.002	213.95	−5.731	<0.001	−0.012, −0.006
Rehabilitation^a^ × Session	−0.033	0.005	229.95	−7.199	<0.001	−0.042, 0.024

SE, standard error; df, degrees of freedom; CI, confidence interval.

^a^ Reference: group without VR.

Individual SPEV values and regression curves, showing a non-linear pattern of compensation, are displayed in Figure 3 for both groups of patients.

#### Survival analysis

**Figure 4:**
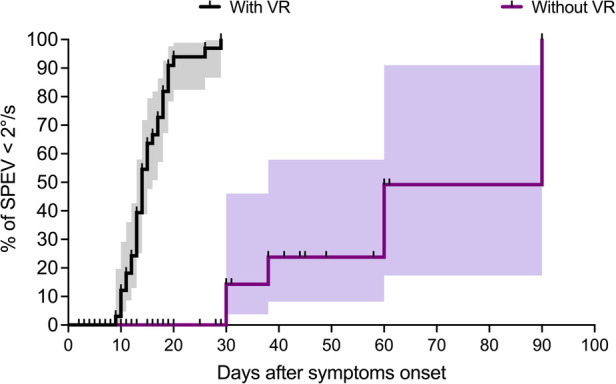
Survival analysis in patients with and without VR (Study 1). Survival analysis with the Kaplan–Meier method shows the percentage of patients with non-significant SN (i.e., SPEV < 2°/s) as a function of the time elapsed after the symptom onset. The graphs (staircase ± 95% CI) show faster recovery in the group with VR compared to the group without VR, with median for SN normalization of 14 days and 90 days for the two groups, respectively.

Figure 4 shows the pattern of change in the percentage of patients from VR and non-VR groups towards non-significant SN (i.e., SPEV < 2°/s). The statistical comparison of the survival curves indicates a significant effect of group (Log-rank test: *χ*^2^_(1) _= 62.99, *p *< 0.0001) with shorter median time for SN normalization for patients receiving VR compared to patients without. This was 14 days and 90 days, respectively. The estimated hazard ratio (logrank) was 14.6 (95% CI: 7.35–29.02), indicating that the rate of SN normalization (SPEV < 2°/s) in patients with early VR was about 15 times the rate of SN normalization in patients with a “natural” compensation of SN.

### Study 2: time course of the SN reduction with early vs. late VR

#### Descriptive analysis of the individual curves of SN over time

**Figure 5:**
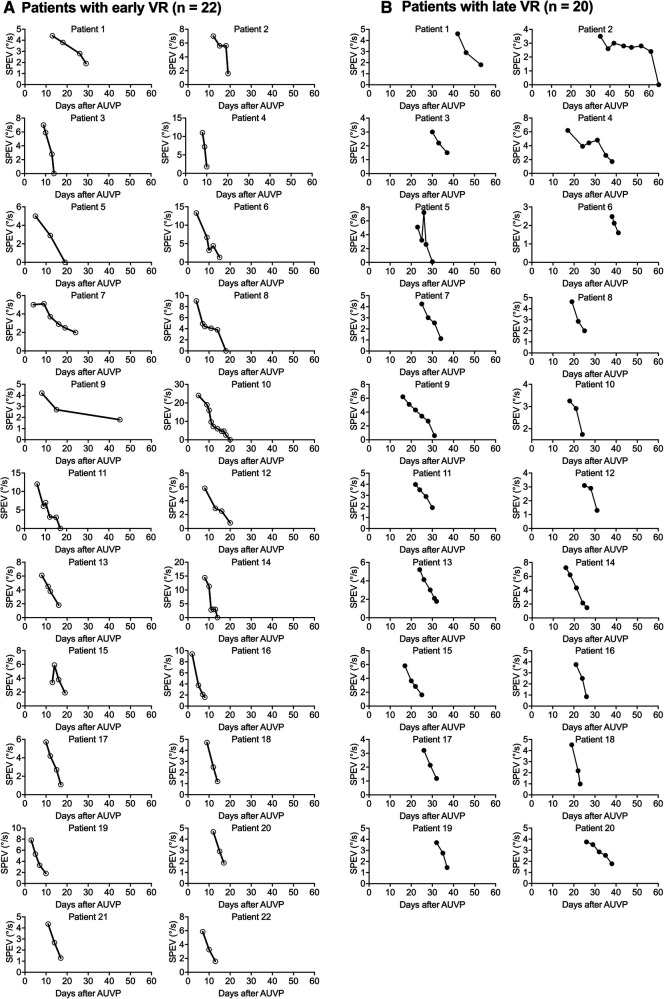
Individual curves of the SPEV of the SN over time after early and late VR (Study 2). SPEV of the SN as a function of days after symptoms onset for each of the patients who underwent and early VR (**A**) or a late VR (**B**) with the unidirectional rotation paradigm.

Figure 5 shows the evolution of the SPEV in 22 patients who underwent early VR (Figure 5A), and in 20 patients who underwent late VR (Figure 5B). All patients regained non pathological SN (SPEV < 2.0°/s) with similar time courses across the two groups: 3–10 VR sessions were necessary for patients with early VR, while 3–8 sessions were necessary for patients with late VR.

The SPEV was significantly reduced as early as the end of the first VR session in both groups. In patients with early VR, the SPEV decreased from 9.1 ± 4.5°/s before VR to 3.3 ± 2.2°/s after the first VR session (36% reduction). Six out of 22 patients (28%) had a SPEV below 2°/s, and two (9%) showed a reversed SN beating to the lesioned side. After the fifth VR session all patients with early VR showed a SPEV below 2°/s.

Similar changes were observed in the late VR group. The mean SPEV decreased from 4.7 ± 1.2°/s before VR to 1.1 ± 0.6°/s after VR (23% reduction), 35% had a SPEV below 2°/s, and 20% showed a reversed SN after the first VR session. All patients with late VR showed a SPEV below 2°/s after the fourth VR session.

We also note that almost all patients in both groups had a higher SPEV at the beginning of the following VR session compared to the SPEV recorded at the end of the previous VR session. However, this was lower than the SPEV measured at the beginning of the previous VR session. This typically characterises a habituation process.

#### Linear mixed-effects model

As participants from the early VR and late VR groups started the VR at very different times post symptoms onset, we analyzed changes in SPEV as a function of the days since the patients started the first VR session. Table 4 shows the parameters estimates for the fixed effects.

**Table 4:** Estimates of fixed effects for Study 2.

Parameter	*B*	SE	*df*	*t*	*p*	95% CI (lower, upper)
Intercept	0.666	0.035	80.57	19.204	<0.001	0.597, 0.735
Rehabilitation^a^	0.149	0.048	79.05	3.116	0.003	0.054, 0.244
Session	−0.019	0.004	172.47	−4.762	<0.001	−0.026, −0.011
Rehabilitation^a^ × Session	−0.012	0.005	173.97	−2.304	0.022	−0.023, −0.002

SE, standard error; df, degrees of freedom; CI, confidence interval.

^a^ Reference: group with late VR.

The linear mixed-effects model indicated that the log-transformed values of SPEV was significantly modulated by the time elapsed since the first VR session (*B* = −0.019, SE = 0.004, *p* < 0.001). As expected from the natural decay of the SPEV, there was a significant difference of early vs. late VR on the SPEV at time 0 (*B* = 0.149, SE = 0.048, *p* = 0.003). Lower SPEV for patients in the late VR group was likely related to the spontaneous recovery that took place between the time of the symptoms onset and the first VR session. The analysis also showed a significant interaction of Session × Timing of VR (*B* = −0.012, SE = 0.005, *p* = 0.022), indicating different patterns of SPEV change over time for both groups. Comparison of the slope of the fit for the log-transformed SPEV values in the early VR group (−0.031) and late VR group (−0.019) indicated that the recovery was 1.6 time faster in the group of patients who underwent early VR.

**Figure 6:**
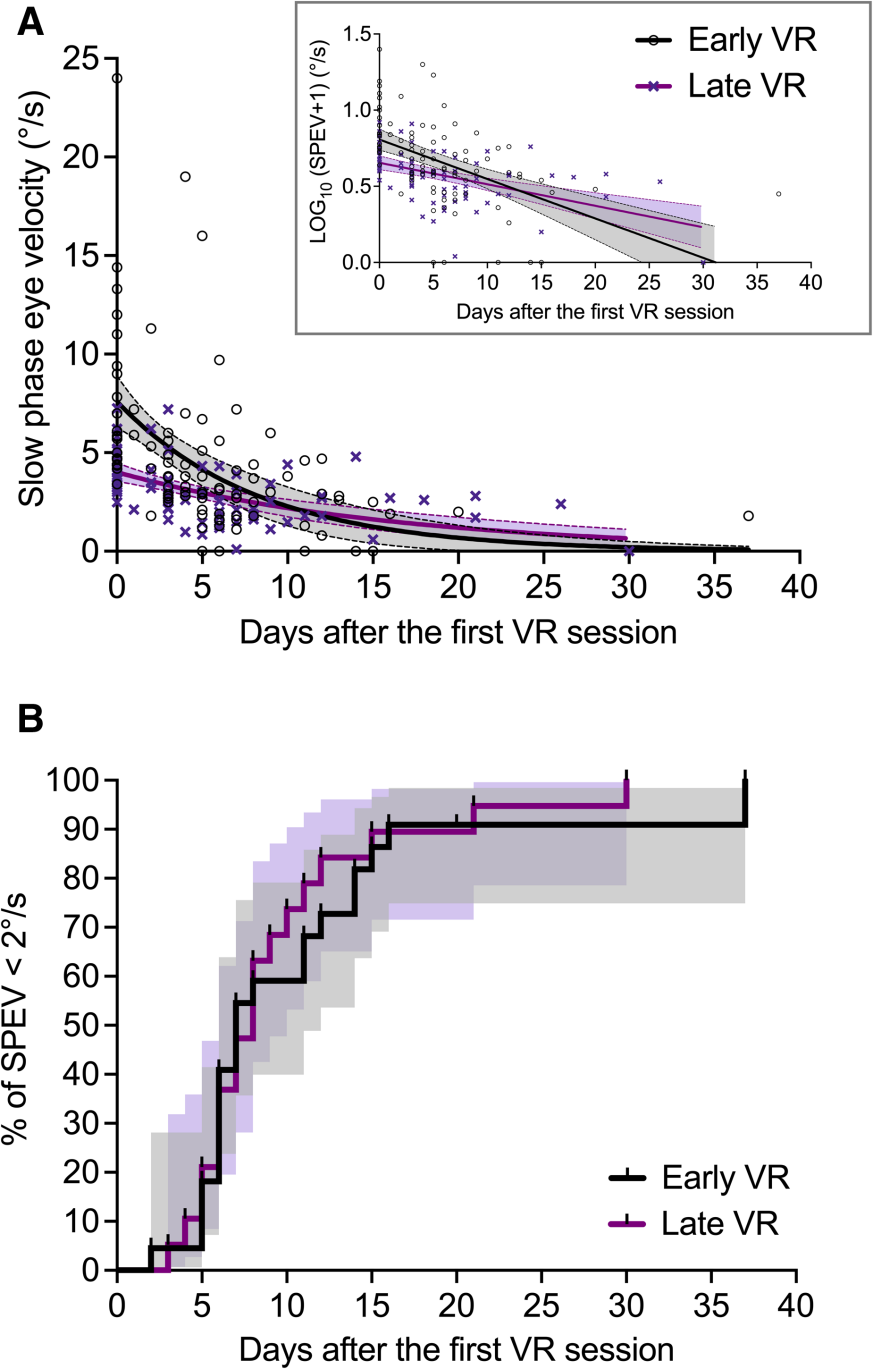
Effect of early and late VR on the evolution of the SPEV of the SN (Study 2). (**A**) SPEV of the SN as a function of the time elapsed since the first VR session (not since symptoms onset), and one-phase decay regression curves for both groups of patients (goodness of fit: *R*^2^ = 0.30 for early VR; *R*^2^ = 0.30 for late VR). The filled areas are the 95% confidence interval of parameters. (**B)** Kaplan–Meier graphs (staircase ± 95% CI) describing the evolution of the percentage of patients with non-significant SN (i.e., SPEV < 2°/s) in the group with early VR and late VR as a function of the time elapsed since the first VR session.

Individual SPEV values and regression curves are shown in Figure 6A for both groups of patients.

#### Survival analysis

Figure 6B shows the Kaplan–Meier graphs describing the evolution of the percentage of patients with non-significant SN (i.e., SPEV < 2°/s) after receiving early or late VR. We found that the survival curves did not differ significantly between the early VR and late VR groups (Log-rank test: *χ*^2^_(1) _= 0.31, *p *= 0.578). The median time for SN normalization was 7 days for patients with early VR and 8 days for patients with late VR. The estimated hazard ratio (logrank) was 1.18 (95% CI: 0.63–2.20), indicating similar rate of SN normalization in patients with early and late VR.

#### Supplementary outcomes

The static and dynamic SVV, as well as the DHI score, have been measured before and after VR in the two groups receiving early and late VR (Table 5). The pre-post VR comparison indicates significant improvement of the static SVV in patients with early VR (*p* < 0.001) and late VR (*p* < 0.02). Before commencing VR, the static SVV deviation was significantly lower in patients in the late VR group when compared to patients in the early VR group (*p* < 0.001). This is possibly due to spontaneous recovery with time. However, both groups regained SVV in the normal range of ±2.5°, and they did not differ significantly after VR.

**Table 5:** Supplementary outcomes of prospective Study 2.

	Early VR		Late VR	
	Before VR	After VR	Before VR	After VR
Static SVV (°)	4.89 ± 2.84	2.10 ± 1.01	2.66 ± 1.73	1.57 ± 1.80
Dynamic SVV (%)	45.8 ± 21.7	35.5 ± 22.9	40.6 ± 23.7	30.2 ± 21.4
DHI (total score)	65.1 ± 16.6	19.1 ± 18.4	54.8 ± 20.1	23.7 ± 17.7

Mean ± SD of the static subjective visual vertical (SVV), the dynamic subjective visual vertical (percentage of directional preponderance), and the dizziness handicap inventory (DHI).

By contrast, the directional preponderance of the dynamic SVV did not improve significantly after VR in both groups of patients. The directional preponderance did not differ significantly between groups neither before VR nor after VR.

Finally, DHI scores were in the moderate range of handicap for both groups before VR, with significantly lower values in patients with late VR compared to patients with early VR (*p* < 0.002). After VR, the total DHI score decreased significantly in both groups of patients (*p* < 0.001), reaching values in the range of weak handicap. The DHI score did not differ significantly between two groups after VR.

## Discussion

The results from Study 1 show that the SN can persist for several months without VR. Study 1 and 2 both show that VR, using a unidirectional rotation paradigm, can significantly improve the time it takes for SN to normalize. Furthermore, SN normalization (SPEV < 2°/s) after VR does not appear to be influenced by the duration since AUVP symptom onset.

### Spontaneous nystagmus reduction with and without vestibular rehabilitation

The static vestibular syndrome in AUVP patients is the combination of impaired unilateral canal and otolith afferents (35–37), inducing perceptual (vertigo, verticality perception), postural (head tilt) and oculomotor (SN, ocular cyclotorsion) deficits. Animal models of unilateral vestibular loss indicate that these deficits result from the strong imbalance between the resting discharge of neurons in the ipsilesional and contralesional vestibular nuclei (3, 4). Animal models also show that the recovery of neurophysiological activity in the vestibular nuclei on the weaker side is a key mechanism to compensate the static syndrome (see Introduction). Furthermore, the timing of static symptoms recovery parallels the recovery of the resting discharge of the vestibular neurons (38). The mechanisms underlying such recovery in AUVP patients remain speculative. Sensory substitutions (2, 10, 21) and functional reorganizations (39) are very likely involved. A voxel-based morphometry study in AUVP patients showed increased gray matter volume in the vestibular nuclei and gracile nucleus on the affected side, as well as in the bilateral middle temporal/V5 area, and increased white matter volume in the pontine commissural vestibular fibers (40). The data suggests that vestibular, somatosensory, and visual inputs are involved in central compensation in AUVP patients.

SN is a static ocular motor sign largely but incompletely recovered up to 1 year after vestibular loss (18, 41). Our data confirm that SN normalization can take several months. AUVP patients without VR, that is, with spontaneous “natural” compensation, showed a non-linear pattern of compensation over time, with a significantly different time course than patients with VR. The survival analysis showed that the median time for SN normalization was significantly longer without VR (90 days without VR vs. 14 days with VR).

Taken together, our data indicate that VR with a unilateral rotation paradigm speeds up the process of SN reduction. We hypothesize that the unidirectional rotations to the weaker side decrease the spontaneous firing rate of the vestibular nuclei neurons on the intact side which, in turn, results in a disinhibition of the affected side by way of the commissural system (Figure 1). Electrophysiological modifications of the commissural field potentials were found after hemilabyrinthectomy in the frog, with an increased number of commissural excitatory postsynaptic potentials and a decreased number of commissural inhibitory postsynaptic potentials (42), and voxel-based morphometry showed white matter changes in the commissural pathways in AUVP patients (40). These two studies indicate a contribution of the commissural system to central vestibular compensation. Stimulation of remaining intact vestibular afferents on the weaker side is a second mechanism that could promote the restoration of spontaneous activity on this side. Using an anti-synaptophysin antibody as a nerve terminal marker, we reported a synaptic density restoration of 60% three weeks post-lesion [(43): in cats]. Synaptic remodeling in the deafferented vestibular nuclei is likely due to the sprouting of new terminals from remaining intact afferent vestibular fibers. These would then make new synaptic contacts on the deafferented vestibular nuclei neurons. In addition, proliferation of postsynaptic membrane receptors increasing the synaptic weight of multisensory afferents to the vestibular nuclei could also contribute to compensation. Our recent investigations in AUVP patients suggest that a full recovery of dynamic canal function, that is, restoration of normal aVOR gain, could depend on the presence of remaining intact vestibular afferents on the weaker side (30). Changes in the physiological properties of the commissural system (contribution from the intact side), and activation of intact afferents (contribution from the weaker side) are two synergistic mechanisms able to restore the spontaneous firing rate on the weaker side and to rebalance the nuclear activity between the two sides. There is evidence from animal models that such mechanisms of neuroplasticity occur very early after unilateral vestibular loss in the deafferented vestibular nuclei [see (12), for review], and that they are dynamically tuned by early, postlesion stimulation and behavioral experience (Hebbian plasticity).

Although still speculative, these mechanisms could explain the reduction of the aVOR directional preponderance observed after unidirectional rotations in AUVP patients, as previously discussed (30, 34). Thus, the effects of VR with a unidirectional paradigm are in line with the effects of other similar VR paradigms, showing a decrease in the asymmetry of the aVOR gain in macaques with a unilateral labyrinthectomy (44) and a decrease in the aVOR directional preponderance in patients with chronic unilateral vestibular dysfunction (45). However, due to different parameters of rotation, the mechanisms underlying the improvement of the aVOR gain and the normalization of the SN reported here may differ in the various VR paradigms that used unidirectional rotations of the whole body. While vision contributes to the asymmetric changes in the horizontal aVOR gain [see (44)], recovery of SN seems, in part, independent of visual inputs. SN decreases at the same rate in animals kept in the dark or in a lighted environment immediately after unilateral labyrinthectomy (32). A recent meta-analysis of vestibular compensation showed that vision has a limited impact on the increase in intrinsic excitability of ipsilesional vestibular neurons and on the recovery of bilateral symmetry in the vestibular nuclei (46).

### Effect of early and late vestibular rehabilitation on the reduction of spontaneous nystagmus

We have previously provided the first demonstration that early VR results in improved dynamic visual acuity and passive aVOR in AUVP patients (30, 34), confirming the concept of a postlesion critical period described in animal models (see [21]). The first few weeks after symptom onset constitutes an opportune time window during which neuroplasticity in the vestibular nuclei is tuned dynamically by sensorimotor feedback from interactions between patients and their environment.

This critical period appears restricted to the recovery of dynamic vestibular functions. Posture in non-challenging/dynamic conditions (stable support, eyes open or closed) recovered in patients who underwent early and late VR (28, 47), whereas dynamic balance (unstable support, eyes closed or with altered visual motion cues) recovered more quickly in patients with early VR (29). Several sensory signals can substitute the lost vestibular inputs to recover static vestibular functions, whereas the recovery of dynamic functions necessitates the full contribution of both labyrinths and/or requires spared vestibular functions from the affected side [see (30)].

The present data provide mixed findings about the effects of early and late VR on the SN. While the linear mixed-effects model revealed a significant interaction of Session × Timing of VR on the SPEV, the survival analysis showed similar percentage of patients with SPEV below 2.0°/s over time and similar median times for SN normalization in both groups.

In line with results from the survival analysis for the SN, the analysis of the static SVV and the DHI score showed a similar recovery timeframe in AUVP patients who underwent early or late VR. By contrast, the dynamic SVV remained uncompensated in both groups of patients. The change in ocular cyclotorsion over time follows closely that of the static SVV (18, 48), and both oculomotor and perceptual deficits recover slowly in parallel over time (49, 50). The ocular cyclotorsion has not been recorded in the present study, but previous investigations showed ocular cyclotorsion up to 3–6 months after a unilateral vestibular neurectomy (17). We also found that ocular cyclotorsion decreased in parallel with both the static SVV and the SN (51). Assuming that static SVV deviation is a static otolithic deficit caused by the neural imbalance between the vestibular nuclei (18), our data suggest that early or late VR can reduce the recovery time course of all static symptoms, including SN, ocular cyclotorsion and deviation of the SVV. By contrast, the directional preponderance of the dynamic SVV did not improve over time in patients who underwent early and late VR. This confirms that abnormal dynamic SVV is potentially a long-term impairment—even a permanent deficit—of visual-otolithic integration after unilateral vestibular loss (15).

The DHI score decreased after VR in all patients, who shifted from moderate to weak handicap. Differences in DHI score larger than 18 points represent a significant change in the patient's handicap (52). Here, we found a much larger reduction of the DHI score after early VR (47 points on average) and late VR (31 points on average). However, as the DHI score is a subjective measure that often does not correlate with objective measures of vestibular functions (53), the decrease in the DHI score reported in the present may not be solely due to the effects of VR.

### Clinical considerations

The results of our studies confirm that the natural reduction of the SN in patients with AUVP is a rather slow process. In some patients, we show a 90-day process of natural normalization of SN. However, some studies have shown that compensation of static postural, oculomotor and perceptual deficits can take up to one year. Of key significance for patients, our data shows the potential for unilateral rotational VR to significantly reduce the normalization time of SN. In addition, we found clinically significant improvements in DHI. Both of these results indicate that VR could positively impact patients' quality of life, and significantly quicker than a naturalistic process. SN normalization is fairly stable once achieved, a supplementary argument in favor of VR with the rotation paradigm or other protocols aimed to reduce the vestibular asymmetries.

## Data Availability

The raw data supporting the conclusions of this article will be made available by the authors, without undue reservation.
